# A Phase I Trial of the Dual MET Kinase/OCT-2 Inhibitor OMO-1 in Metastatic Solid Malignancies Including MET Exon 14 Mutated Lung Cancer

**DOI:** 10.1093/oncolo/oyad146

**Published:** 2023-06-01

**Authors:** Melinda A Pruis, Matthew G Krebs, Ruth Plummer, Filip De Vos, Eric Angevin, Hans Prenen, Martin D Forster, Glen Clack, Annegret Van der Aa, Marc Tjwa, Ellen Jansen, Timothy Perera, Martijn P Lolkema

**Affiliations:** Department of Oncology, Erasmus MC Cancer Institute, Rotterdam, The Netherlands; Division of Cancer Sciences, Faculty of Biology, Medicine and Health, The University of Manchester and The Christie NHS Foundation Trust, Manchester Academic Health Science Centre, Manchester, UK; Oncology Department, Newcastle University and Newcastle Hospitals NHS Foundation Trust, Newcastle, UK; Department Medical Oncology, University Medical Center Utrecht, University Utrecht, Utrecht, The Netherlands; Drug Development Department (DITEP), Gustave Roussy Institute, Villejuif, France; Department of Medical Oncology, University Hospital Antwerp, Antwerp, Belgium; Department of Oncology, UCL Cancer Institute/ University College London Hospitals NHS Foundation Trust, London, UK; Octimet Oncology NV, Belgium; Octimet Oncology NV, Belgium; Octimet Oncology NV, Belgium; Octimet Oncology NV, Belgium; Department of Oncology, Erasmus MC Cancer Institute, Rotterdam, The Netherlands; Octimet Oncology NV, Belgium; Department of Oncology, Erasmus MC Cancer Institute, Rotterdam, The Netherlands

**Keywords:** OMO-1, MET, cancer, NSCLC, exon 14

## Abstract

**Introduction:**

Targeted therapy in non-small cell lung cancer (NSCLC) patients with mesenchymal epithelial transition (MET) exon 14 skipping mutations (METex14) and MET amplifications has improved patients’ outcomes. The development of more potent MET kinase inhibitors could further benefit these patients. The aim of this trial is to determine the safety and recommended phase 2 dose (RP2D) of OMO-1 (an oral dual MET kinase/OCT-2 inhibitor) and to assess preliminary clinical efficacy in METex14-positive NSCLC and other MET-positive solid tumors.

**Materials and Methods:**

This was a first-in-patient, open-label, multicenter study of OMO-1 in patients with locally advanced or metastatic solid malignancies. A standard 3 + 3 dose escalation design was utilized starting at a dose level of 100 mg BID continuously. Preliminary efficacy was investigated in patients with METex14-positive NSCLC, and MET amplified NSCLC and other solid tumors (MET basket).

**Results:**

In the dose-escalation part, 24 patients were included in 5 dose levels ranging from 100 mg twice daily (BID) to 400 mg BID. Most common adverse events (≥ 20%) were nausea, fatigue, vomiting, increased blood creatinine, and headache. The RP2D was determined at 250 mg BID. In the expansion cohorts, 15 patients were included (10 in METex14-positive NSCLC cohort and 5 in MET basket cohort) and received either 200 or 250 mg BID. Eight out of the 10 patients with METex14 positive NSCLC had stable disease as the best response.

**Conclusion:**

OMO-1 was tolerated at the dose of 250 mg BID and shows initial signs of MET inhibition and anti-tumor activity in METex14 mutated NSCLC patients.

## Teaser

Targeted therapy for non-small cell lung cancer (NSCLC) with mesenchymal epithelial transition (MET) exon 14 skipping mutations and MET amplifications have improved outcomes. This study aimed to determine the safety and recommended phase II dose of OMO-1 to assess preliminary clinical efficacy in METex14-positive NSCLC and other MET-positive solid tumors.

**Implications for Practice**

The oral dual MET kinase/OCT-2 inhibitor OMO-1 has an acceptable safety profile at doses up to 250 mg BID and shows preliminary clinical efficacy in pre-selected patients with MET exon 14 skipping mutated lung cancer. The potential immune-modulatory effect of OCT-2 inhibition could be particularly interesting in this population of patients with MET-mutated lung cancer because of the MET-induced immunosuppressive tumor-microenvironment.

## Introduction

Dysfunction of the mesenchymal epithelial transition (MET) tyrosine kinase receptor and the MET pathway through *MET* amplifications or *MET* mutations are associated with the development of several solid tumors.^1^ The therapeutic strategy of inhibiting the MET pathway in MET-addicted solid tumors has been mostly best researched in NSCLC. In NSCLC, 4%-10% of the tumors are considered to be MET dependent^2^; in patients with NSCLC*MET* exon 14 skipping (METex14) mutations are present in 3%-5%, and high-level *MET* amplifications are present in 1% and 4.1% depending on the definition (MET/Centromere ratio (MET ratio) ≥5 and MET gene copy number (GCN) ≥5, respectively, by in situ hybridization).^3-7^*MET* amplifications are also described as a resistance mechanism to EGFR TKIs in *EGFR*-positive NSCLC.^8^ Targeted therapy with MET tyrosine kinase inhibitors (TKIs) has led to improved outcomes in patients with advanced METex14 mutated NSCLC.^2,9^ MET inhibitors with higher affinity for MET, eg, capmatinib and tepotinib, seem to perform better than less selective inhibitors.^9,10^ Sensitivity of MET amplified NSCLC to MET TKIs is more heterogeneous, both in the de novo and in the acquired resistance setting.^10-13^

Besides its role in tumorigenesis, the MET pathway is known to have immune regulatory functions, amongst others through the upregulation of indoleamine-2,3-dioxygenase (IDO), an immunosuppressive enzyme. IDO catalyzes the degradation of tryptophan to kynurenin. Depletion of tryptophan leads to decreased differentiation of immune cells, while the metabolic intermediate kynurenin inhibits immune cell function.^14^ Therefore MET-induced IDO upregulation in the tumor leads to an immunosuppressive tumor microenvironment (TME).^15-17^ Besides increased IDO expression, patients with *MET*-positive NSCLC display a higher tumor mutational burden (TMB) and PD-L1 expression than *MET* wild-type NSCLC resulting in possible benefit from immune checkpoint inhibitors (ICI) in contrast to *EGFR* mutated NSCLC.^18-20^ The combination of targeted therapy with an immunomodulating agent could have a synergic anti-tumor effect on MET-driven NSCLC.

OMO-1 is a highly potent, orally bioavailable dual MET kinase/OCT-2 inhibitor. In preclinical studies, OMO-1 leads to complete inhibition of tumor growth in diverse in vivo MET-activated tumor models.^21^ In addition, in EGFR TKI-resistant models with *MET* amplification, the combination of erlotinib and OMO-1 induced tumor regression.^21^ In addition to MET inhibition, OMO-1 was identified as a potent inhibitor of the organic cation transporter 2 (OCT-2), which is involved in the active secretion of creatinine and tryptophan.^22^ The suppressed OCT-2 mediated secretion of tryptophan might lead to increased plasma levels of tryptophan, thereby stimulating T-cell proliferation and differentiation.

Here we describe a phase I study to establish the safety and preliminary efficacy of the dual MET kinase/OCT-2 inhibitor OMO-1 in patients with solid tumors refractory to standard treatment and, subsequently, in patients with MET-positive solid tumors.

Trial registration: Clinicaltrials.gov registry number NCT03138083.

## Methods

This study was part of a modular, first-in-patient, openlabel multicenter study of OMO-1, administered orally, mono-therapy, and in combination with EGFR-TKI, in patients with locally advanced, unresectable, or metastatic solid malignancies (study protocol can be). OMO-1 is a small molecule inhibitor of MET. Module 1 (OMO-1 monotherapy) consisted of Part A (dose escalation) and Part B (expansion cohorts). Part A assessed the safety and tolerability of ascending doses of OMO-1 given as monotherapy in unselected patients with locally advanced, unresectable, or metastatic malignancies and determined the recommended phase 2 dose (RP2D). Part B assessed the preliminary efficacy of the RP2D of OMO-1 in patients with METex14-positive NSCLC (cohort 1) and patients with MET amplified NSCLC or patients with other MET positive solid tumors, either METex14 or MET amplification (MET basket; cohort 2). The MET status of the tumor was assessed locally by site-specific guidelines. For MET exon 14 skipping mutations next generation sequencing (NGS) was used and for MET amplifications either NGS or fluorescence in situ hybridization (Supplementary 1). Prior treatment with a MET inhibitor was an exclusion criterion. Module 2 (OMO-1 in combination with EGFR TKI) investigated the preliminary efficacy of OMO-1 in patients with activating EGFR mutated NSCLC and an acquired MET amplification after EGFR TKI treatment. The study was conducted at 8 clinical centers in the UK, the Netherlands, Belgium, and France and was performed in accordance with the Declaration of Helsinki and the principles of Good Clinical Practice. The protocol was approved by the Oxford C Research Ethics Committee (Ref 17/SC/0229) and by all local ethical committees of the participating sites. All patients provided written informed consent before undergoing any study procedures.

The primary objective of the study was to determine the safety and tolerability of OMO-1 when given orally to patients with locally advanced, unresectable, or metastatic solid malignancies and to define the dose and schedule for further clinical evaluation. Secondary objectives were to assess the preliminary efficacy of OMO-1 by response evaluation criteria in solid tumors (RECIST) v 1.1 and to characterize the pharmacokinetics (PK) and pharmacodynamics (PD) of OMO-1, following a single dose and/or at steady state after multiple dosing, when given orally as monotherapy or in combination with epidermal growth factor receptor (EGFR)-tyrosine kinases inhibitor (TKI).

Eligible patients were aged ≥18 years with Eastern Cooperative Oncology Group (ECOG) performance score 0-1, adequate organ function, histological, or cytological confirmation of locally advanced, unresectable, or metastatic solid malignancy refractory to conventional treatment. Exclusion criteria included a diagnosis of diabetes mellitus or current use of metformin (OMO-1 might increase exposure to metformin because metformin is secreted by OCT-2), prior splenectomy, active illness or viral infection, symptomatic CNS metastases, a history of epilepsy or uveitis, abnormal urinary function or outflow obstruction, and previous bone marrow transplant or recent surgery.

OMO-1 was administered orally, twice daily (BID) with a minimal interval of 4-5 h with food in a continuous 21 day/cycle regimen until clinical progression or emergence of significant toxicity. The starting dose was 100 mg BID. The dose was escalated sequentially in cohorts of at least 3 patients using a standard 3 + 3 dose escalation design. Dose-escalation stopped when significant safety or tolerability concerns were observed in 2 or more patients and where the non-optimal long-term exposure dose (OLED) was exceeded. The RP2D was defined as the maximum dose whereby the safety or tolerability findings did not exceed 1 out of 6 patients. Computed tomography (CT) based tumor assessments were conducted according to RECISTv1.1 at screening and every 6 weeks once study treatment had commenced. Safety assessments were continually assessed and based on all adverse events (AEs; graded according to the National Cancer Institute Common Terminology Criteria for Adverse Events version 4.0, 2009 (NCI-CTCAE v 4.0)), clinical laboratory data, and physical examinations. In view of the association between MET inhibition and ocular toxicity, ophthalmological examination was conducted at baseline.^23^ In addition to urea and creatinine, tests of renal function such as serum Cystatin C and urinary KIM1 were conducted at baseline and on day 1 of subsequent treatment cycles. During the monotherapy dose escalation Module 1 Part A, blood samples were collected for PK analysis throughout the study as per protocol (S1). To explore the effects of the OCT-2 inhibition plasma levels of tryptophan and kynurenine were sampled. The difference in mean tryptophan plasma levels before and after OMO-1 exposure was statistically tested with a paired *t*-test (if data is normally distributed) or a Wilcoxon signed rank test (if not normally distributed).

The dose-limiting toxicity (DLT) evaluation period was within the first cycle of treatment; although all events beyond this point were also reviewed at the time of dose escalation to ensure an OLED was chosen as RP2D. A DLT was defined as any of the following occurring during the DLT-evaluation period: grade 3 or 4 non-hematological toxicity (excluding nausea/vomiting/diarrhea that responded to standard medical treatment), grade 4 hematological toxicity, febrile neutropenia, grade 3 thrombocytopenia with bleeding, QTc prolongation (>500 ms, or 60 ms above baseline), any toxicity related to OMO-1 that was clinically significant or unacceptable or unresolved after a treatment delay of >14 days and any toxicity clinically significant and/or unacceptable, greater than that at baseline and judged to be a DLT by the SRC.

### Safety and Preliminary Efficacy Analyses

Safety analyses were descriptive. With respect to primary objectives and endpoints, no specific hypotheses were tested statistically. The primary focus was on determining the safety profile and RP2D, and the identification of a range of biologically active doses and PK of OMO-1 in patients with cancer. Safety, PK, and PD biomarker data were reviewed on an ongoing basis in line with the cohort progression criteria by the Safety Review Committee (SRC). Preliminary efficacy of OMO-1 by RECIST 1.1. was assessed locally. The best overall percentage change in tumor size is defined as the percentage change from baseline value that represents the largest decrease or smallest increase (data as provided by local assessment). The clinical benefit rate is derived as the proportion of patients with a partial response or a duration of stable disease (SD) of at least 8 weeks. Initially, the design in Module 1 Part A included parallel cohorts of patients with MET-positive tumors per dose level. Eventually, these patients were analyzed in Part B in expansion cohorts. Therefore, patients in Part B have received either 200 mg or 250 mg BID.

### Pharmacokinetics and Pharmacodynamics

Analysis was performed using PKSolver to define *C*_max_, trough concentration (*C*_trough_), AUC_∞_, AUC_0-last_, *V*_d_, clearance, elimination half-life (*t*_½_), and *T*_max_ of OMO-1.^24^ In the paired biopsy analysis, expression of total MET and phosphorylated MET was performed by immunohistochemistry.

## Results

**Figure 1.**
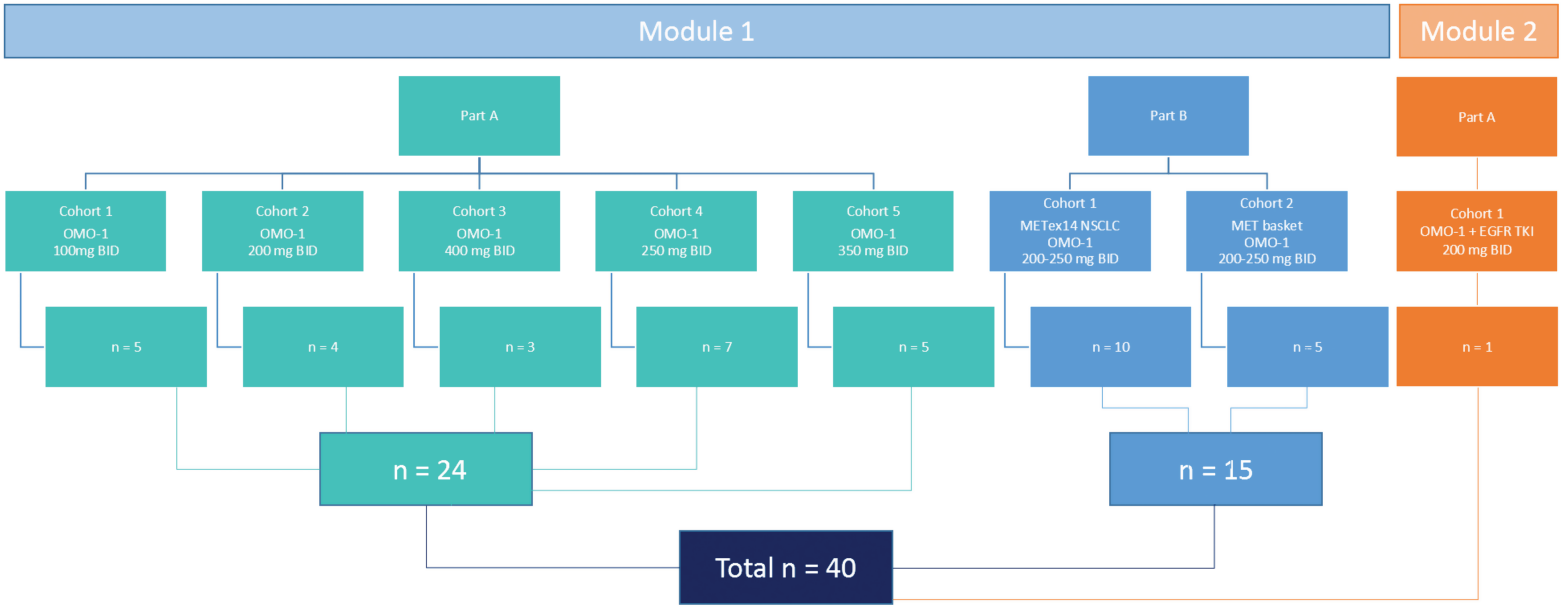
Overview of study design and patient inclusion. Dose-escalation was performed in Module 1, Part A. Cohort expansion in MET positive subgroups was performed in Module 1, Part B and Module 2, Part A. BID, twice daily, EGFR TKI, epidermal growth factor receptor tyrosine kinase inhibitor, METex14, MET exon 14 skipping mutation, NSCLC, non-small cell lung cancer.

Between August 08, 2017 and December 09, 2019 a total of 40 patients were enrolled in the study. The primary analysis cutoff date was 25 May 2020 and the final cutoff date was 05 August 2020. At the time of data cutoff, no patients remained in study. In the dose-escalation part (module 1, part A) 24 patients were included in 5 dose levels: 100 mg BID, 200 mg BID, 400 mg BID, 250 mg BID, and 350 mg BID (Fig. 1). In the expansion cohorts (module 1, part B) 15 patients were included: 10 patients with METex14-positive NSCLC in cohort 1 and 5 patients with MET positive solid tumors in cohort 2 (Table 1). Patients in Part B received either 200 mg BID or 250 mg BID. No patients in these cohorts was treated with MET inhibitor prior to inclusion, as this was an exclusion criterion. Recruitment closed early due to a strategic decision (including lack of funding), therefore one patient was included in Module 2 Part A, OMO-1 combined with an EGFR TKI, at a dose level of 200 mg BID. Patient characteristics are described in Table 1.

**Table 1.** Patient characteristics per cohort.

	Module 1							Module 2
	Part A					Part B		Part A
Number of patients, *n* (%)	Cohort 1 OMO-1 100 mg BID *N* = 5	Cohort 2 OMO-1 200 mg BID *N* = 4	Cohort 3 OMO-1 400 mg BID *N* = 3	Cohort 4 OMO-1 250 mg BID *N* = 7	Cohort 5 OMO-1 350 mg BID *N* = 5	Cohort 1 OMO-1 METex14 NSCLC 200-250 mg BID *N* = 10	Cohort 2 OMO-1 MET basket 200-250 mg BID *N* = 5	Cohort 1 OMO-1 + EGFR TKI 200 mg BID *N* = 1
Median age in years (range)	64.0 (51-74)	69.5 (51-74)	65.0 (64-66)	75.0 (42-84)	58.0 (48-74)	68.5 (56-80)	57.0 (44-67)	55
Sex (%)								
Male	4 (80%)	3 (75%)	3 (100%)	3 (43%)	3 (60%)	5 (50%)	4 (80%)	0
Female	1 (20%)	1 (25%)	0 (0%)	4 (57%)	2 (40%)	5 (50%)	1 (20%)	1 (100%)
ECOG performance status at screening								
0	2 (40%)	2 (50%)	1 (33%)	2 (29%)	2 (40%)	2 (20%)	1 (20%)	1 (100%)
1	3 (60%	2 (50%)	2 (67%)	5 (71%)	3 (60%)	8 (80%)	4 (80%)	0
Cancer type								
Colorectal	3 (60.0%)	1 (25.0%)	2 (66.7%)	1 (14.3%)	3 (60.0%)	0	1 (20%)	0
GI	0	2 (50.0%)	0	2 (28.6%)	2 (40.0%)	0	1 (20%)	0
NSCLC	0	1 (25.0%)	0	1 (14.3%)	0	10 (100%)	1 (20%)	1 (100%)
Other	2 (40.0%)	0	1 (33.3%)	3 (42.9%)	0	0	2 (40%)	0
Median number of lines of prior chemotherapy (range)	2.0 (0-4)	2.5 (1-3)	3.0 (2-6)	2.0 (2-6)	4.0 (2-6)	2 (1-6)	7 (1-9)	0
MET aberration								
METex14 (NSCLC)						10 (100%)	—	—
METex14 (other)						—	1 (20%)	—
METAmp	—	—	—	—	—	—	4 (80%)	1 (100%)
≥ 10 GCN							0	
≥5-≤ 10 GCN							2 (50%)	
Unknown							2 (50%)	

Abbreviations: BID, twice daily, GCN, gene copy number, GI, gastrointestinal, METex14, MET exon 14 skipping mutation, METamp, MET amplification, NSCLC, non-small cell lung cancer.

### Pharmacokinetic Profile

**Figure 2.**
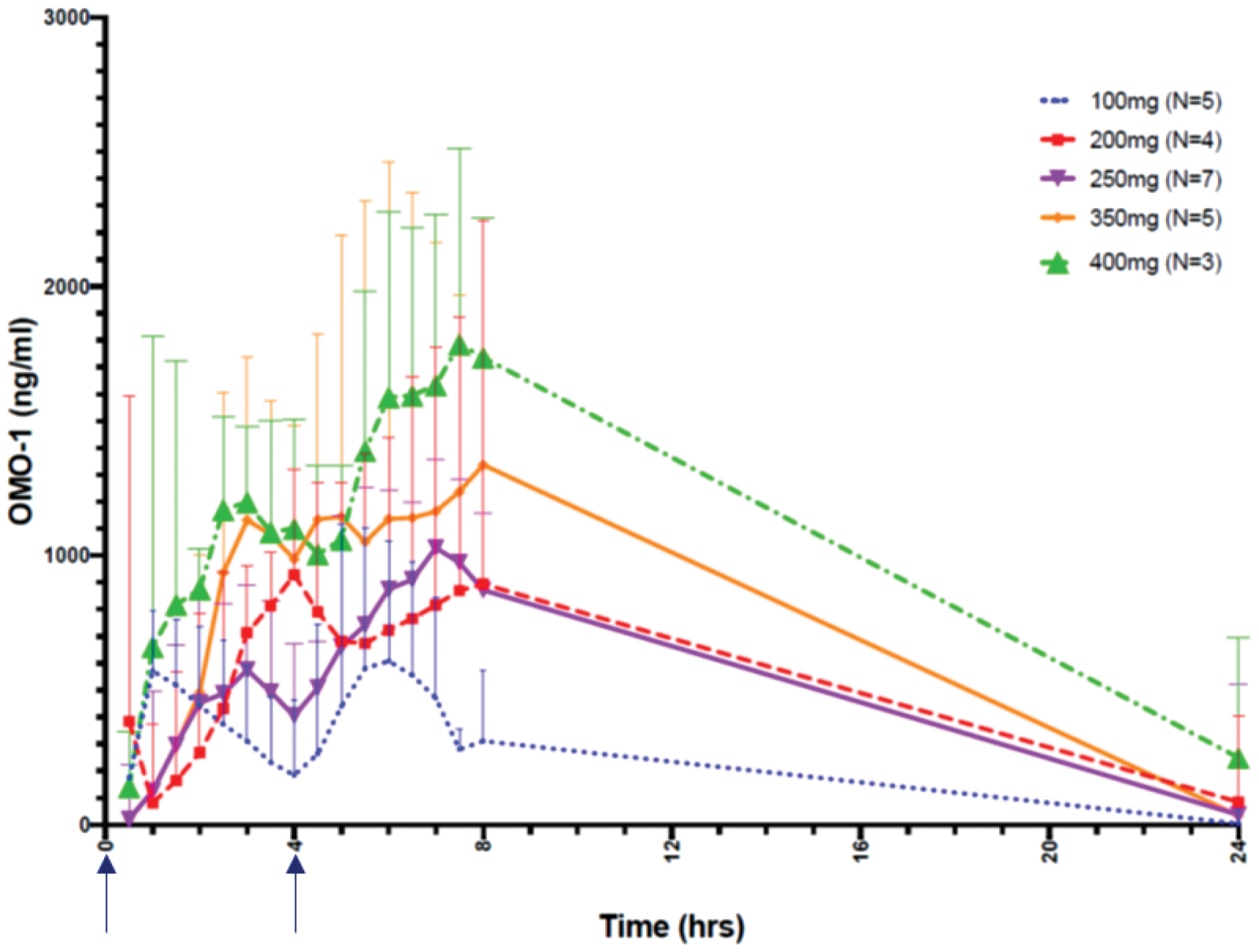
Geometric mean (SD) plasma concentration-time profiles of OMO-1 on cycle 1 day 1 in Module 1 Part A. The arrows on the X-axis indicate dosing times at 0 and 4 h.

OMO-1 has a *T*_½_ of 2.5-3 h and plasma exposure as measured by AUC_(0-8 h)_ is dose-proportional without accumulation (Fig. 2). OMO-1 is generally rapidly absorbed and the median *T*_max_ was relatively similar across the dose escalation cohorts [range 5-7.8 h]. The second daily dose of OMO-1 was taken between 4 and 5 h after the first dose. This led to a second peak in exposure and a *T*_max_ longer than the *t*_½_. At RP2D of 250 mg BID, *C*_max_ was 1372 ng/mL, and AUC_(0-8 h)_ was 5684 h ng/mL.

### Dose Escalation

In cohorts 1 (100 mg BID) and 2 (200 mg BID) no DLTs or other safety concerns were seen as concluded by the SRC. In cohort 3 (400 mg BID) also no DLTs were reported, but 2 out of 3 patients required a dose reduction due to treatment-related toxicity and 6 TEAEs led to OMO-1 treatment discontinuation in 2 patients (66.7%). In one of these 2 patients, the TEAEs leading to discontinuation were drug-related nausea, increased bilirubin, increased creatinine, and diarrhea (increased stoma output) (CTCAE grade 2). The other patient was discontinued due to hypersensitivity to OMO-1 (CTCAE grade 2; retching and tremor after administration) after a previous dose reduction due to drug-related flu-like symptoms (CTCAE grade 3). Although these TEAES were not reported as formal DLTs, further treatment at that dose level was precluded as decided by the SRC. In addition, the planned dose level of 600 mg BID was replaced by a lower dose level of 250 mg BID (cohort 4). No DLTs or significant safety concerns were seen in cohort 4. Cohort 5 (350 mg BID) was the final dose-escalation. However, the nature of the AEs reported in the 400 mg BID and 350 mg BID, along with the high rate of TEAEs leading to withdrawal resulted in the SRC deciding that these doses were not in keeping with long term dosing; therefore, 250 mg BID was chosen as the monotherapy RP2D.

### Analysis of Treatment Emergent Adverse Events in Module 1 (Monotherapy) Part A (Dose Escalation)

In 23 out of 24 patients in Module 1 Part A (monotherapy, dose-escalation), 129 drug-related TEAE were recorded. The most common drug-related TEAE (≥ 20%) were nausea (11 patients, 46%), fatigue (10 patients, 42%), vomiting (9 patients, 38%), increased blood creatinine (9 patients, 38%), and headache (5 patients, 21%) (Table 2). A higher proportion of patients in the 200 mg, 400 mg, and 250 mg BID cohorts (3 patients [75.0%], 3 patients [100%], and 3 patients [42.9%], respectively) had increased blood creatinine values than in the 100 mg and 350 mg BID cohorts (no events for either cohort). Fatigue was recorded for a higher proportion of patients in the 400, 250, and 350 mg BID cohorts (2 patients [66.7%], 3 patients [42.9%], and 3 patients [60.0%], respectively) than in the 100 mg and 200 mg BID cohorts (1 patient in both cohorts [20.0% and 25.5%, respectively]).

**Table 2.** Most common drug-related AEs in order of frequency in Module 1 Part A.

CTCAE (v4.03) preferred term	100 mg BID *n* = 5			200 mg BID *n* = 4			250 mg BID *n* = 7			350 mg BID *n* = 5			400 mg BID *n* = 3			All doses *n* = 24		
	Drug-related TEAEs																	
	All grades	Grade ≥3	SAE	All grades	Grade ≥3	SAE	All grades	Grade ≥3	SAE	All grades	Grade ≥3	SAE	All grades	Grade ≥3	SAE	All grades	Grade ≥3	SAE
Nausea	2 (40%)	1 (20%)	1 (20%)	3 (75%)	—	—	4 (57%)	—	1 (14%)	1 (20%)	—	—	1 (33%)	—	1 (33%)	11 (45%)	1 (4%)	3 (13%)
Fatigue	1 (20%)	—	—	1 (25%)	—	—	3 (43%)	1 (14%)	—	3 (60%)	—	—	2 (67%)	—	—	10 (42%)	1 (4%)	—
Vomiting	1 (20%)	1 (20%)	1 (20%)	2 (50%)	1 (25%)	—	3 (43%)	—	—	3 (60%)	—	1 (20%)	—	—	—	9 (38%)	2 (8%)	2 (8%)
Blood creatinine ↑	—	—	—	3 (75%)	—	—	3 (43%)	1 (14%)	—	—	—	—	3 (100%)	—	1 (33%)	9 (38%)	1 (4%)	1 (4%)
Headache	1 (20%)	—	—	3 (75%)	—	—	—	—	—	1 (20%)	—	—	—	—	—	5 (21%)	—	—
Dizziness	1 (20%)	—	—	1 (25%)	—	—	1 (14%)	—	—	1 (20%)	—	—	—	—	—	4 (17%)	—	—
Diarrhoea	—	—	—	2 (50%)	—	—	1 (14%)	—	—	—	—	—	1 (33%)	—	1 (33%)	4 (17%	—	1 (4%)
Anemia	—	—	—	2 (50%)	1 (25%)	—	1 (14%)	—	—	—	—	—	1 (33%)	—	—	4 (17%)	1 (4%)	—
Decreased appetite	1 (20%)	—	—	1 (25%)	—	—	1 (14%)	—	—	1 (20%)	—	—	0	—	—	4 (17%)	—	—
Tachycardia	—	—	—	—	—	—	1 (14%)	—	—	1 (20%)	—	—	1 (33%)	—	—	3 (13%)	—	—
Chills/ influenza like symptoms	—	—	—	1 (25%)	—	1 (25%)	—	—	—	1 (20%)	—	—	1 (33%)	1 (33%)	1 (33%)	3 (13%)	1 (4%)	2 (8%)
Blood bilirubin ↑	—	—	—	—	—	—	—	—	—	—	—	—	1	—	1 (33%)	1 (4%)	—	1 (4%)
Neutrophil count↓	—	—	—	—	—	—	—	—	—	—	—	—	1	—	1 (33%)	1 (4%)	—	1 (4%)
Total *n* with AE	5 (100%)	2 (40%)	2 (40%)	4 (100%)	1 (25%)	1 (25%)	7 (100%)	2 (28%)	1 (14%)	4 (80%)	—	1 (20%)	3 (100%)	1 (33%)	2 (67%)	23 (96%)	6 (25%)	7 (29%)

In total, 11 drug-related SAEs were reported in 7 patients. Nausea and vomiting were reported as drug-related SAEs in more than 1 patient (in 3 and 2 patients respectively).

No grade 5 (fatal) TEAEs were recorded.

**Figure 3.**
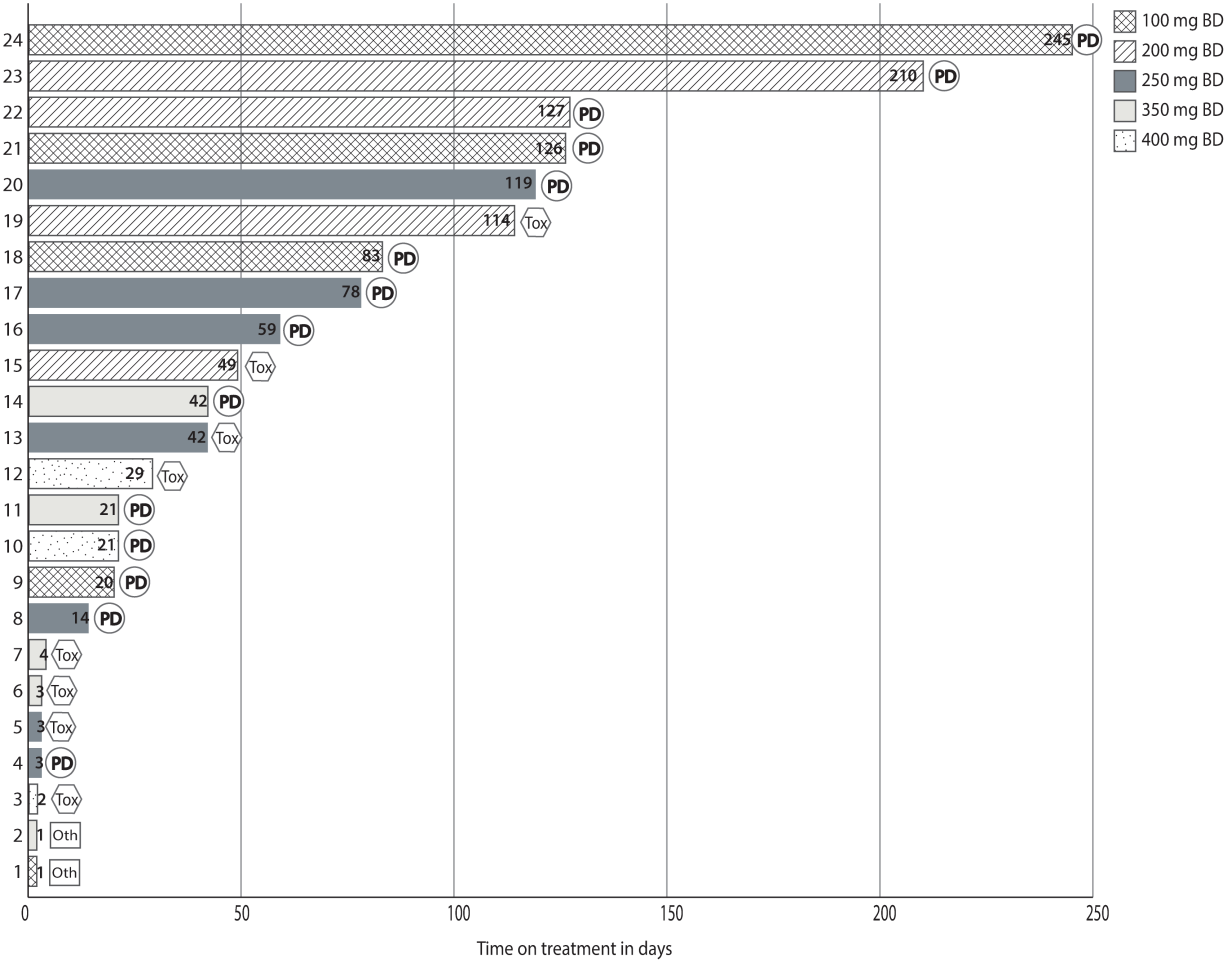
Time on treatment of patients in Module 1, Part A (dose-escalation) in days and reasons for discontinuation. PD, progressive disease, including RECIST v.1.1. confirmed progressive disease and clinical evidence of progressive disease, tox, drug-related toxicity leading to discontinuation, oth, other reason for discontinuation of treatment.

Six patients were on treatment with OMO-1 for more than 100 days of which 2 patients exceeded 200 days (Fig. 3). All these patients received either 100 mg, 200 mg, or 250 mg BID. In these lowest dose levels (100, 200, and 250 mg BID), 25% (4/16) of the patients were discontinued due to related toxicity. In the 350 mg BID and 400 mg BID cohort, half of the patients had to discontinue due to related toxicity (4/8). In total, 8 patients were discontinued due to toxicity related to OMO-1 (Fig. 3). Fatigue, vomiting, and nausea were recorded for more than 1 patient. One patient was discontinued due to hypersensitivity to OMO-1 (grade 2 in severity, related to IMP). Other reasons for discontinuation were disease progression, withdrawal of consent, and incorrectly initiated on OMO-1 (did not meet inclusion criteria as patient had diabetes). The toxicity profile of patients in Part B was similar to Part A for Module 1 (OMO-1 monotherapy) and the single patient in Module 2.

### Pharmacodynamics

**Figure 4.**
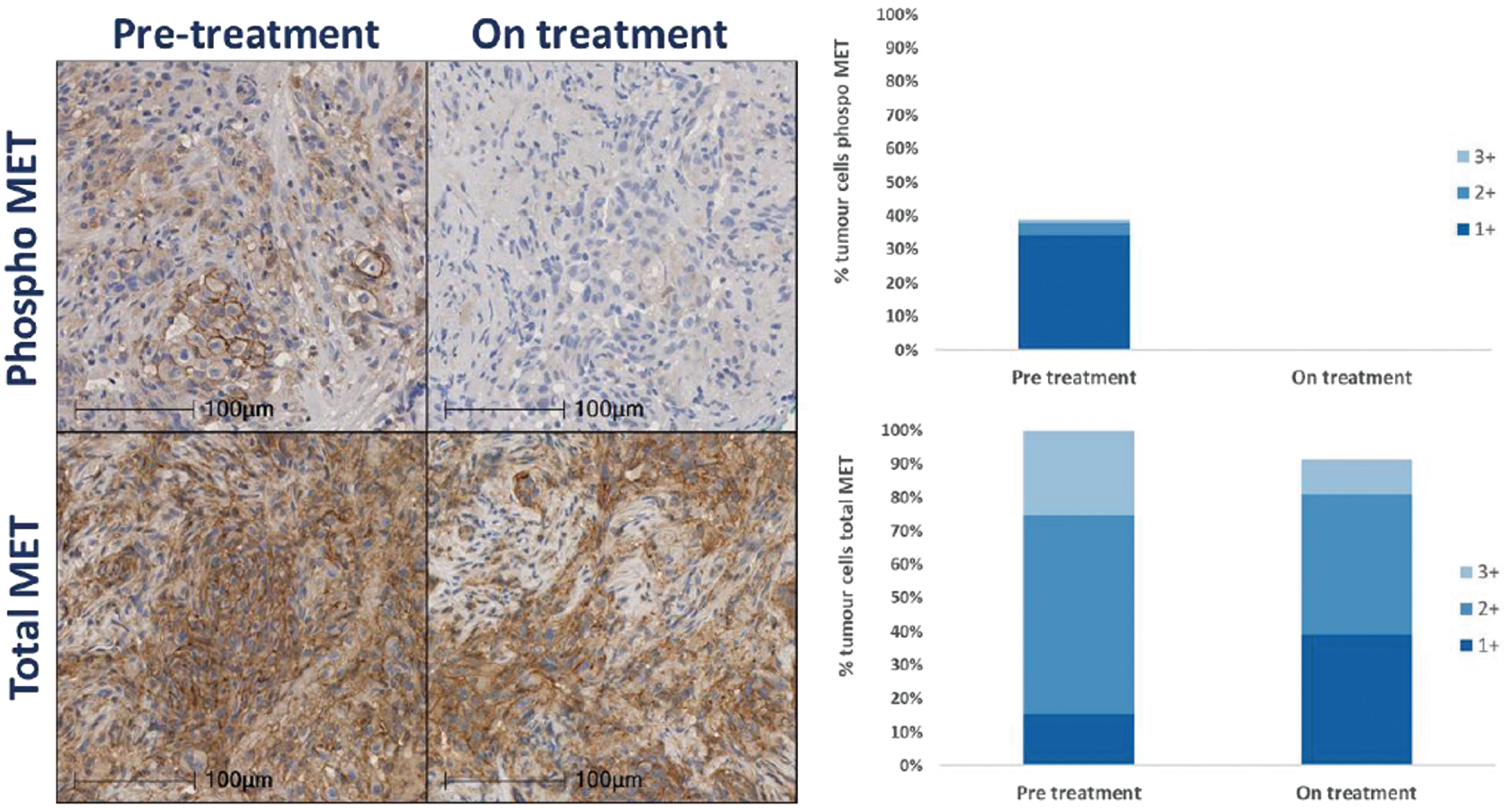
Total and phosphorylated MET assessment with immunohistochemistry on paired biopsy samples of a patient with MET exon 14 mutated non-small cell lung cancer dosed at 200 mg BID.

Paired biopsy analysis was achieved for one patient with NSCLC with a MET exon 14 skipping mutation, dosed with 200 mg BID. The on-treatment biopsy showed a near-complete inhibition of phosphorylated MET, without affecting total MET levels (Fig. 4).

Mean creatinine levels were higher than baseline for all patients in all cohorts at all time points measured throughout the study (ranging from 6.6% to 121.5% increase from baseline), in keeping with on-target OCT-2 inhibition; however, throughout the study, overall mean creatinine levels were within the normal ranges and changes in mean creatinine levels over time were lowest in cohort 1 (100 mg BID).

Plasma tryptophan and kynurenin levels on cycle day 1 at pre-dose and 8 h first post-dose were available for 30 patients in Module 1. A significant increase in mean tryptophan plasma levels on cycle 1 day 1 was seen between pre-dose samples and 8 h post-dose samples (9475 ng/mL vs. 10 605 ng/mL, 95% CI [247.622, 2011.871], *P* = .014). The median kynurenin level in the pre-dose sample (509 ng/mL, interquartile range (IQR) 396-624) slightly decreased in the 8 h post-dose sample (471 ng/mL IQR 396-559), but significance was not reached (*P* = .57).

A decrease in mean kyn/trp ratio was seen between pre-dose samples and 8 h post-dose samples (57 vs. 49 resp, CI 95% [4.1738-11.3506], *P* < .001). The changes in plasma tryptophan levels and kyn/trp ratio were not dose or *C*_max_ dependent.

### Preliminary Efficacy of OMO-1 as Monotherapy and Combined With EGFR TKI

**Figure 5.**
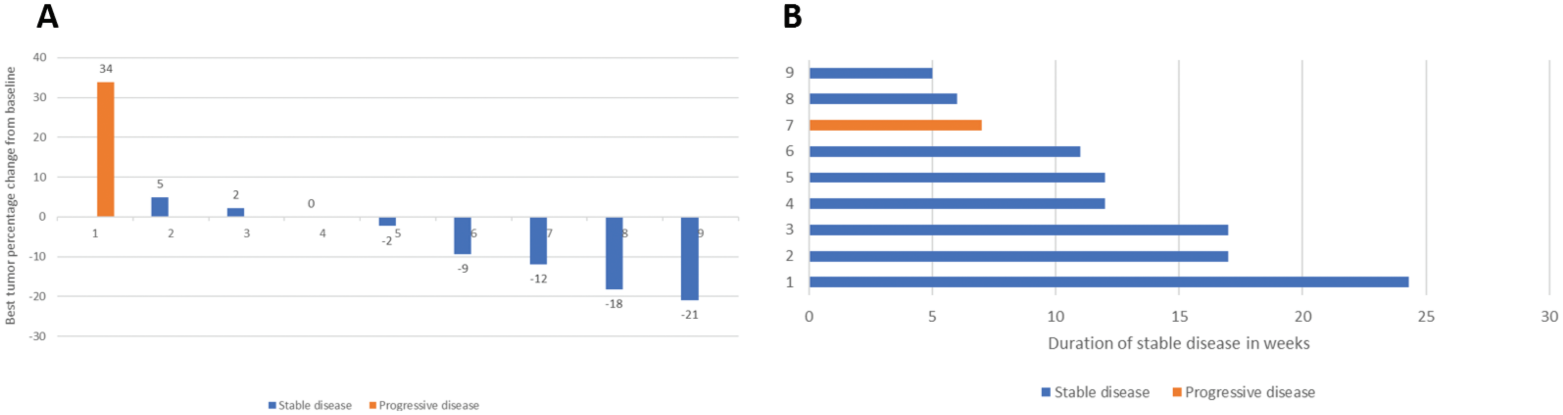
Response to OMO-1 of MET exon 14 mutated NSCLC cohort. **A**. Best response in tumor percentage change from baseline. **B**. Duration of stable disease in weeks.

In the METex14 NSCLC expansion cohort, 10 patients were treated with OMO-1 monotherapy. Nine patients had at least one response evaluation by imaging. The majority of the patients had stable disease as the best response (8/9 patients) (Fig. 5A). No patients had a partial response. A clinical benefit duration of at least 8 weeks was recorded for half of the patients (5/10 patients) (Fig. 5B). In patients in the basket expansion cohort (MET basket), 4 patients were included with a MET amplified tumor. MET amplification status was known in 2 patients by local assessment (sequencing and in situ hybridization): 8 copies and >7 copies resp (S2). One patient had a MET exon 14 skipping mutated cholangiocarcinoma. No clinical benefit of any length was recorded (5/5 patients) in this cohort.

One patient was treated in Module 2 (combination with EGFR TKI); a 55-year-old female, with an EGFR-positive stage 4 NSCLC who had progressed on gefitinib. Tumor biopsy at progression showed an acquired MET amplification for which the patient was included in Module 2. Patient was treated with 200 mg BID of OMO-1 in combination with gefinitib (EGFR TKI). OMO-1 was interrupted due to serum Cystatin C increase of >1.5 fold from baseline, considered related to OMO-1. OMO-1 was restarted at one dose level lower (100 mg BID). Best response by local assessment was stable disease and best response by central assessment was partial response. Treatment was discontinued in week 23 due to disease progression.

## Discussion

In this phase I clinical trial, we found that OMO-1 had an acceptable long-term safety profile at 250 mg BID. PD analysis of a paired biopsy of a patient with METex14 mutated NSCLC showed near-complete inhibition of phosphorylated MET. Moreover, we observed modest clinical efficacy of OMO-1 in patients with METex14 mutated NSCLC. The development of more highly selective MET inhibitors is crucial for optimal treatment of MET exon 14 skipping mutation-positive NSCLC patients.^25^

In the dose-escalation cohorts, OMO-1 was tolerated in the majority of the patients on the lower dose-levels (100, 200, and 250 mg BID). As discussed in the dose-escalation results section, 400 mg BID and 350 mg BID were deemed not suitable for long-term dosing. The safety profile of OMO-1 is mostly comparable to other MET TKIs, but more patients on OMO-1 had to discontinue treatment due to toxicity than with capmatinib and tepotinib (also in the lower dose cohorts).^9,10^ Also, more gastrointestinal toxicity was seen in patients on OMO-1 (250 mg BD, all grades; nausea—57%, vomiting—43%, diarrhea—14%), than with capmatinib (all grades; nausea—46%, vomiting—26%, diarrhea—17%), and tepotinib (all grades; nausea—26%, vomiting—6%, diarrhea—22%). The mechanism behind the difference in toxicity is unknown. Another important drug-related TEAE was increased blood creatinine which is due to the competitive inhibition of creatinine secretion by OCT2. In the phase I trial of OMO-1 in healthy human volunteers no renal toxicity was observed, suggesting that increased blood creatinine does not translate into kidney injury during OMO-1 treatment.^26^ In this trial, additional markers for renal function to reliably evaluate possible kidney injury, eg, cystatin C and KIM-1, did also not identify any significant renal function issues in monotherapy OMO-1. This on-target effect on blood creatinine levels was notable, but mostly mild and overall did not cause any issues with OMO-1 administration. In contrast, the predecessor compound of OMO-1, JNJ-38877605, had renal toxic properties, which precluded it from further development.^27^ No reports were made of peripheral edema related to OMO-1, which is described as a common AE for MET inhibitors.^9,10,28^ The mechanism behind MET inhibition-related peripheral edema is yet to be elucidated, so it is unclear why OMO-1 does not cause edema.

As theorized, the mean plasma tryptophan level increased 8 h after OMO-1 administration and the mean kynurenin/tryptophan ratio decreased. This supports the hypothesis that OMO-1 inhibits the active secretion of tryptophan through OCT-2. In some patients, tryptophan levels decreased which could be due to low drug exposure, but we could not confirm this. As tryptophan is an amino acid present in most protein-based foods, food intake could be a confounding factor. Validation of these results in a larger cohort with dietary monitoring would be needed to confirm this hypothesis.

Following the dose-escalation part, the efficacy of OMO-1 was investigated in selected expansion cohorts; METex14 mutated NSCLC, other MET amplified, or MET mutated solid tumors (basket cohort). In METex14 mutated NSCLC, OMO-1 showed modest efficacy as almost all patients had stable disease as best response and no partial response (RECIST v.1.1) was recorded. One of the reasons for this low response rate could be the prior therapy status of the patients. Most patients had received prior anti-cancer therapy, which could have induced molecular heterogeneity and instability leading to accelerated development of resistance mechanisms. This negative effect of prior therapy has also been seen in trials with other MET TKIs.^10^ However, no patients were treated with a MET TKI prior to OMO-1. Additionally, in 40% of the patients a co-occurring mutation was present potentially causing intrinsic resistance.^29^ Other new MET TKIs, like capmatinib and tepotinib, have shown impressive results in METex14 mutated NSCLC with object response rates of 68% (95% CI: 48, 84) to capmatinib and 43% (95% CI: 32%, 56%) to tepotinib with a median response duration of respectively 12.6 months (95% CI: 5.5, 25.3) and 10.8 months (95% CI: 6.9, not estimable).^9,10^ Both drugs have also received accelerated Food and Drug Administration (FDA) approval for the treatment of metastasized NSCLC. In the MET basket cohort, no clinical efficacy was seen. We believe this can be attributed to the level of MET amplification in the tumors, as none of the patients had a tumor with a high-level MET amplification (≥10 GCN). The most recent research shows that only high-level MET amplifications should be recognized as driver events and are therefore susceptible to targeted therapy.^10^ This is substantiated by the fact that low to medium MET amplified tumors (<10 GCN) are less sensitive to MET TKIs than high MET amplified tumors.^10^ In addition to the difference in response, high-level MET amplifications seem to be mutually exclusive with other known driver mutations, whereas low-level amplified tumors often contain a co-occurring driver mutation.^30^ At the time of trial design, the stratification of MET amplification was not yet common practice and therefore this was not part of the inclusion criteria. In further research with MET TKIs it would be recommended to enrich for high-level MET amplified solid tumors. Unfortunately, there is still no gold standard definition of high-level MET amplification and this also differs per technique used.^31^ There are signals that MET amplified NSCLC might be sensitive to immune checkpoint inhibitors, however only limited and retrospective research has been done.^32,33^ Therefore, compounds like OMO-1 combining potential immunomodulatory effects with targeted therapy could be an interesting mode of therapy for this group, although the actual immunomodulatory effects of OMO-1 in vivo remain unclear. Because OMO-1 will not be further developed for this indication, no additional insights can be gained.

The combination of OMO-1 and an EGFR TKI in EGFR mutated and MET-amplified NSCLC (Module 2) was investigated after the dose-escalation part in Module 1. As the trial was closed prematurely due to a strategic decision (including lack of funding), only one patient was included in this combination cohort in Module 2. After failure on an EGFR TKI, OMO-1 was able to bypass the resistance to the EGFR TKI, resulting in a partial response with a treatment benefit of more than 5 months, which is comparable to similar trials.^34,35^

In conclusion, OMO-1 is safe and well tolerated to administer at doses up to 250 mg BID and shows preliminary clinical efficacy in pre-selected patients with METex14 mutated NSCLC.

## Acknowledgments

We thank the participating patient and site staff for their participation and contributions to this study. M.W. acknowledges support from National Institute for Health Research (NIHR) Manchester Biomedical Research Centre, NIHR Manchester Clinical Research Facility at The Christie and Manchester Experimental Cancer Medicine Centre (Manchester, UK). Manchester, Newcastle and UCL clinical sites acknowledge support from CRUK/DOH England as Experimental Cancer Medicine Centres.

## Supplementary Material

oyad146_suppl_Supplementary_Material

## Data Availability

The data underlying this article will be shared on reasonable request to the corresponding author.
