# Supplementary material for: A Phase I Trial of the Dual MET Kinase/OCT-2 Inhibitor OMO-1 in Metastatic Solid Malignancies Including MET Exon 14 Mutated Lung Cancer
Source: Oncologist. 2023 Jun 1;28(12):e1248–58. doi: 10.1093/oncolo/oyad146 (PMC10712729; doi:10.1093/oncolo/oyad146)
Supplement: oyad146_suppl_Supplementary_Material [file oyad146_suppl_supplementary_material.docx]

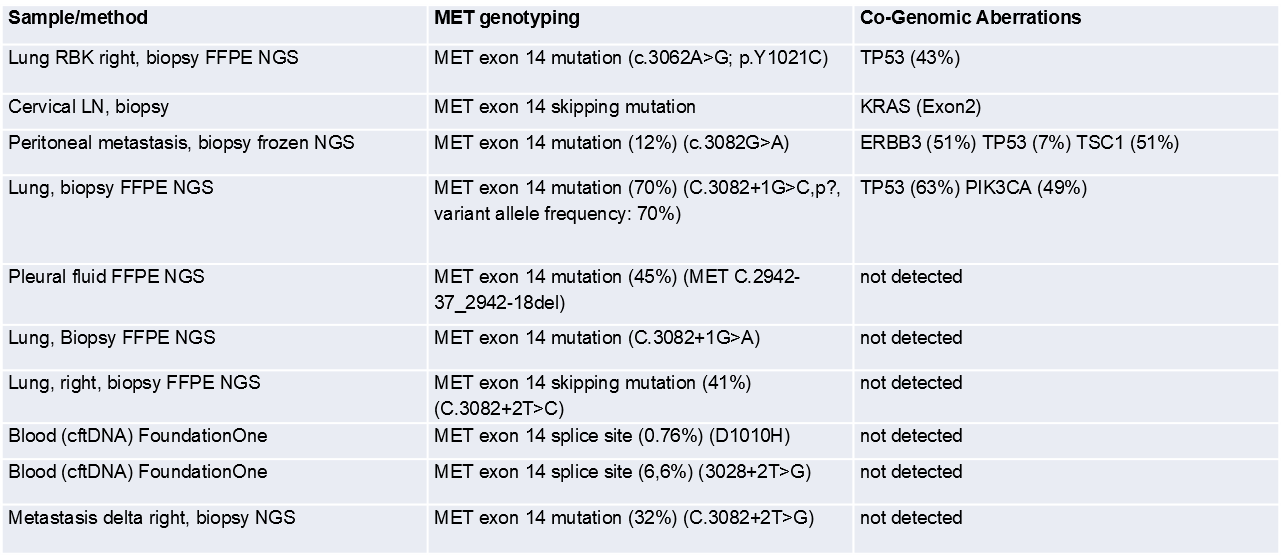

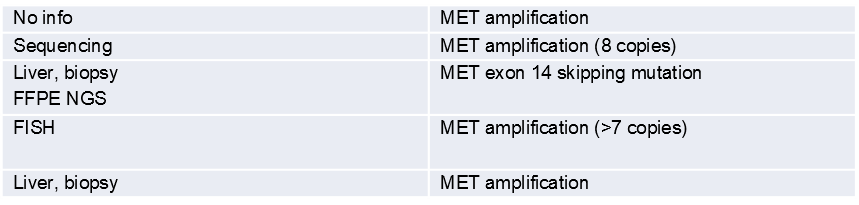


**MET status for subjects enrolled in Module 1 Part B, expansion cohort 2**

**MET status for subjects enrolled in Module 1 Part B, expansion cohort 1**

MET status for subjects enrolled in Module 2

| **Sample/method** | **MET genotyping** | **Co-Genomic Aberrations** |
| --- | --- | --- |
| LN, punction NGS and SISH | 9,36 fold MET amplification | TP53 (exon 8) (5%) **EGFR (exon 21) (72%)** TP53 (Exon 7) (18%) |
